# The gender gap among school children in poor rural areas of western China: evidence from a multi-province dataset

**DOI:** 10.1186/s12939-016-0442-5

**Published:** 2016-09-29

**Authors:** Hua Zhou, Di Mo, Chengchao Zhou, Alexis Medina, Yaojiang Shi, Linxiu Zhang, Scott Rozelle

**Affiliations:** 1 Schools of Economics & Management, Tongji University, Shanghai, China; 2 Freeman Spogli Institute for International Studies, Stanford University, Stanford, USA; 3 School of Public Health, Shandong University, Jinan, China; 4 Center for Chinese Agricultural Policy, IGSNRR, Chinese Academy of Sciences, Beijing, China; 5 Center for Experimental Economics in Education, Shaanxi Normal University, Xi’an, China

## Abstract

**Background:**

The gender gap remains a major impediment in the path towards equality and it is especially wide in low-income countries. Up to the early 2000s, many studies documented extensive inequalities in China: girls had poorer health, less nutrition and less education than their male counterparts. The goal of this study is to examine whether the gender gap persists, given that China is now making the transition into the ranks of upper-middle income countries. We consider educational outcomes, mental and physical health status, as well as non-cognitive outcomes.

**Methods:**

We draw on a dataset containing 69,565 observations constructed by combining data from 7 different school-level surveys spanning 5 provinces. The surveys were all conducted by the authors between 2008 and 2013 using uniform survey instruments and data collection protocols in randomly selected schools across western provinces in rural China. The sample children range in age from 9 to 14 years (with 79 % of the sample being aged 10 to 12). Our analysis compares rural girls with rural boys in terms of 13 different indicators.

**Results:**

With the exception of anemia rates, the health outcomes of girls are equal to those of boys. Girls and boys are statistically identical in terms of weight-for-age, height-for-age, and prevalence of intestinal worm infections. Girls performed better than boys on five of six cognitive and educational performance indicators. Girls performed worse than boys on all mental health indicators. All estimates are robust to the inclusion of different age ranges, controlling for the level of household assets, ethnic minority status, as well as the addition of provincial dummies.

**Conclusions:**

Our findings suggest that with the exception of non-cognitive outcomes, anemia and standardized math test scores, the gender gap in our study areas in China appears to be diminishing.

## Background

The plight of girls in low-income countries—in terms of both health and education—has drawn attention from many researchers in a variety of fields. Empirical evidence has shown that girls in low-income countries often have worse health outcomes than their male counterparts. For example, excessive female mortality at young ages has been well documented in low-income countries such as India [1–4], China [5–8], Pakistan [9], and Bangladesh [10]. Even though the under-five mortality rates are decreasing in most developing countries, girls are not benefiting proportionally from these declines [8]. The rate of infection with intestinal worms (*Ascaris lumbricoides*) in Laos is higher for girls aged 12–23 months than for boys of the same age (30 % versus 24 %) [11]. Similar gender gaps have been found with Giardia infections in Zambian preschools [12] and with soil-transmitted helminths (STHs) in the National Primary School of Malaysia [13].

Besides having worse health, girls have also been shown to have worse nutrition and education outcomes. Data from Brazil, the Gaza strip, and India all show a higher prevalence of anemia among girls than boys [14–16]. Among eighth grade students in Nepal, boys have higher mean math test scores compared with girls (13.1 versus 10.1) [17].

When countries become richer, however, this gender gap seems to close; the literature shows that in developed countries, the health, nutrition, and educational outcomes of girls are typically better than or equal to those of boys. For example, in the Netherlands, research shows no statistical difference between boys and girls (aged 7 to 12 years) with respect to height (HAZ), weight (WAZ), or Body Mass Index (BMI) [18]. Other researchers show that the BMI of girls is similar to or better than that of boys in other countries in Western Europe, including Portugal [19, 20], the UK [21], Wales [22], and the United States [23]. In terms of educational performance and attainment, girls in developed countries are often found to outperform boys. For example, girls have been shown to have higher levels of mathematics achievement in Hong Kong [24], and to have higher language scores in Finland [25] and the Netherlands [26].

Although many studies in developed countries show that gender gaps in education, health and nutrition seem to draw close as income levels rise, similar results have not been found in the case of mental health problems. For example, many studies in developed countries have found that adolescent girls are significantly more likely to experience low and moderate levels of depression, anxiety, and low self-esteem than adolescent boys [27–30]. Fewer studies have been conducted in developing countries. However, a study in Egypt, for example, found that Egyptian girls are twice as likely to suffer from depression as boys [28].

From the 1980s through the early 2000s, when China was still counted among the ranks of poor and lower middle-income countries, researchers found significant signs that young Chinese girls (aged 0–17 years) were not doing as well as their male peers [29–32]. In 2006-07, researchers found that boys still had higher mean hemoglobin (Hb) levels relative to girls (ages 12–14) [32], and the prevalence of iron-deficiency anemia was significantly higher among high school girls than among high school boys (23.4 % versus 17.2 %) [33]. The average school enrollment rate is higher for boys than for girls among rural children in both the 7–14 year age range and the 15–18 year age range [34].

One possible explanation for this gender gap in China may be cultural. There is a strong literature base showing that rural China has traditionally valued sons over daughters. To the extent that this is still persistent, parents—especially those in poorer households—might be more likely to allocate limited resources to the more valued children—in this case, sons [35, 36]. As a result, boys may be expected to have better health and educational outcomes than girls.

Over the past decade, since 2005, China has joined the ranks of middle-income countries and household incomes have risen accordingly [37]. During 2008 and 2013, China has also issued several national policies, such as the Free Lunch Scheme across the poor rural areas and the New Cooperative Medical Scheme [38, 39]. Though such policies are yet to be rigorously examined, it is plausible that they can slowly contribute to narrowing the gender gap in China. Have girls “caught up” with boys in terms of health, nutrition, and educational outcomes? The evidence is mixed. On the one hand, some studies suggest that the gender gap is closing. In 2006 and 2007, one study found that WAZ measures of girls were similar to those of boys [40]. Another study in the same year found no significant gender differences in underweight preschoolers (aged 0 to 5 years old) [41].

On the other hand, since 2005, other research has documented a persistent gender gap. For example, Ren and Rammohan (2014) find that the gender gap in WAZ widened between 1991 to 2009 [42], while Chyi and Zhou (2014) find that primary and junior high school enrollment rates are still higher among boys than among girls [43]. In addition, there is evidence to suggest that girls still suffer from mental health issues disproportionately more than boys do [44, 45], although this research has not typically been based on empirical measures.

Unfortunately, nearly all of the available evidence since 2005 is based on either small sample sizes or cover a small geographical region, so it is difficult to draw any firm conclusions from the existing literature.

In this study, we aim to fill this gap in the literature. The goal of our study is to describe the health and nutrition status, the cognitive and educational performance, and the non-cognitive outcomes of girls, ages 9 to 14, in rural areas of western China and to compare their outcomes to those of their male counterparts. To do this, we draw on a dataset containing nearly 70,000 observations constructed by combining data from several of our own school-level surveys. Using these data, we are able to compare rural girls with rural boys in terms of 13 different health, nutrition, cognition, educational performance, and non-cognitive outcome variables.

## Methods

### Data

**Fig. 1:**
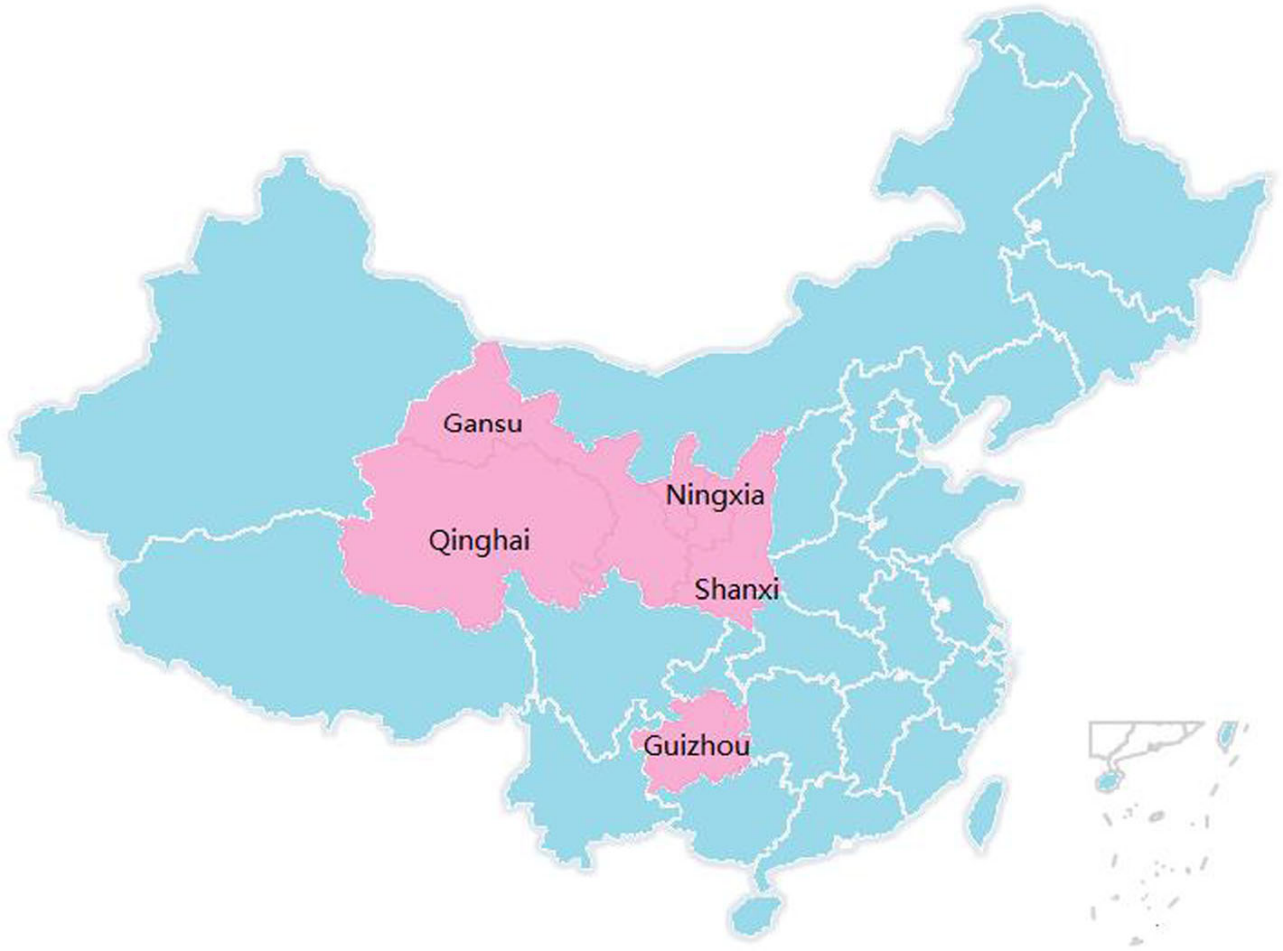
Location of five study provinces in China

The data used for this study are aggregated from seven different school-level surveys that the authors and collaborators conducted between 2008 and 2013. Figure 1 shows the location of the five provinces covered by the dataset: Ningxia, Qinghai, Gansu, Shaanxi and Guizhou province. The provinces that are included in our sample are relatively poor provinces in China. The average GDP per capita (PPP adjusted) is $4,209 for Gansu, $11,736 for Qinghai, $12,469 for Ningxia, $13,636 for Shaanxi and $8,481 for Guizhou. These provinces all have a lower GDP per capita than the national average of $13,980 [46]. Table 1 provides the provinces, years, sample sizes and primary outcomes of the surveys that the paper uses.

**Table 1:** Description of surveys and datasets

(1)	(2)	(3)	(4)	(5)	(6)
Project number	Province	Year	Sample size	Age	Primary outcome variables
1	Shaanxi	2008	4,055	9–14	Standardized math test score, Math course grades given by teachers, Mental Health Test, Hemoglobin
2	Shaanxi	2009	6,013	9–13	Standardized math test score, Math course grades given by teachers, Mental Health Test, Self-esteem, Self-efficacy, Hemoglobin
3	Ningxia, Qinghai	2009	15,169	9–14	Standardized math test score, Math course grades given by teachers, Mental Health Test, Hemoglobin
4	Gansu, Qinghai, Shaanxi	2011	32,915	9–14	Standardized math test score, Math course grades given by teachers, Standardized Chinese test score, Chinese course Grades given by teachers, Hemoglobin
5	Ningxia	2011	1,800	11	Standardized math test score, Math course grades given by teachers, Mental Health Test, Self-esteem, Hemoglobin
6	Gansu	2010	5,306	9–14	Standardized math test score, Math course grades given by teachers, Mental Health Test, Self-esteem, Self-efficacy, Hemoglobin
7	Guizhou	2013	4,307	9–11	WAZ, HAZ, STH, Standardized math test score, Math course grades given by teachers, Working memory, Processing speed, Hemoglobin

### Sample selection

The seven surveys all employed random sampling strategies that were nearly uniform across studies. First, we obtained a list of all counties in each of the five provinces. Second, we randomly selected sample counties from those meeting our study criteria. Third, using official records, we created a list of all primary (and/or secondary) schools in the sample counties. Fourth, we randomly selected schools from the resulting sampling frame. Finally, within each of the randomly selected schools we randomly selected students (or classes of students) for inclusion in the studies. For more information on the sampling procedure, please refer to Additional file 1.

### Data collection and outcome measures

We use a mega-dataset that is pooled from seven previously collected surveys. Each of the surveys was designed independently to study a particular topic, such as anemia, worms, and educational performance of the rural population in China [44, 47–57]. This explains why some of the age ranges of the participants differ slightly in two of the seven surveys (Table 1). Nevertheless, since most of the surveys measured math test scores, all of the surveys targeted children that were ages 9 to 14 – so that participants are old enough to take the administered standardized tests. In fact, nearly four out of every five students (79 %) of our sample are between the ages of 10 and 12.

Even though each survey initially served as an independent study, the procedures were almost uniform across the seven surveys. Hence, this should serve as a point of strength for the study and its use of the pooled dataset. In total, the dataset includes information on 1005 schools from 71 rural inland counties in the five study provinces. During the baseline survey we collected data on students’ basic demographic information, including gender and age. Table 2 provides definitions for the key variables used in the paper.

**Table 2:** Variable definitions

(1)	(2)	(3)
Variable number	Variable	Description
1	WAZ	Weight-for-age z-score
2	HAZ	Height-for-age z-score
3	Anemia rate	Hb < 115 g/L, if age > =9 and < =11 (1 = yes, 0 = no); Hb < 120 g/L, if age > =12 and < =14 (1 = yes, 0 = no)
4	STH infection	Child is infected with any of the three types of STH: *Ascaris*, hookworm, or *Trichuris* (1 = yes, 0 = no)
5	MHT	Mental Health Test. The purpose of the test was to measure the level of each student’s anxiety. The test is scored out of 90 points, where a lower (higher) score corresponds to lower (higher) anxiety.
6	Self-esteem score	Standardized score on Rosenberg Self-esteem Scales I & II (SES)
7	Self-efficacy score	Standardized score on General Self-efficacy Scale (GSE)
8	Standardized math test score	Math test score that is standardized by subtracting the mean and dividing by standard deviation within each wave of survey of each project.
9	Math course grades given by teachers	Normalized grades given by teachers
10	Standardized Chinese test score	Math test score that is standardized by subtracting the mean and dividing by standard deviation within each wave of survey of each project.
11	Chinese course grades given by teachers	Normalized grades given by teachers
12	Working memory index (WMI)	Standardized score on the working memory module of the WISC-IV
13	Processing speed index (PSI)	Standardized score on the processing speed module of the WISC-IV

The primary outcomes examined in this study include 13 measures of health, nutrition, cognition, educational performance, and non-cognitive outcomes. To measure health and nutrition, we have data on weight-for-age z-scores (WAZ), height-for-age z-scores (HAZ), infection with soil-transmitted helminths (STH), and anemia prevalence. We collected two measures of cognition: working memory, and cognitive processing speed. We collected four measures of educational performance: scores from standardized tests of math and Chinese language, and grades given by teachers in math and Chinese language courses. We collected three measures of non-cognitive outcomes: scores on a Mental Health Test, a self-esteem scale, and a self-efficacy scale.

Children’s height and weight were measured and recorded by trained nurses from local provincial-level hospitals. The children were measured in light clothing without shoes. Weight was measured with a calibrated electronic scale. Body height was measured using a standard tape measure. WAZ scores were calculated using a SAS program for the 2000 Center for Disease Control growth chart for children aged 0–20 years [58]. Physical indicators of height were used to construct HAZ scores using WHO AnthroPlus, a software application of the WHO Reference 2007 for children aged 5 to 19 years old that is used to monitor the growth of school-aged children and adolescents [59].

Data on STH infections were collected from 2179 school-aged children in May, 2013 in Guizhou province. Children were considered positive for STH infection if either one of their stool samples tested positive for one or more types of STHs, including *Ascaris lumbricoides* (Ascaris), *Trichuris trichuria* (whipworm), and *Ancylostoma duodenale* or *Necator americanus* (hookworm).

Hemoglobin concentrations (Hb) were measured onsite using a Hemocue Hb 201+ finger prick system. Per WHO guidelines, we use an Hb cutoff of 115 g/L for children aged 9 to 11 years and 120 g/L for those aged 12 to 14 years [60].

To measure cognition, two cognitive measures were generated using the Wechsler Intelligence Scale for Children, Fourth Edition (WISC-IV): working memory and processing speed [61]. The working memory index (WMI) is assessed through two core subtests: the Digit Span subtest and the Letter Number Sequencing subtest. The processing speed index (PSI) is assessed through two core subtests: the coding subtest and the symbol search subtest. Raw scores obtained from these subtests are converted to age-scaled index scores using tables of norms from the official WISC-IV administration and scoring manual for China.

To measure educational performance, students were administered a standardized Chinese language test and a standardized math test. Our enumeration team monitored the tests, carefully proctored them in order to minimize cheating, and strictly enforced the time limits. Scores on both standardized tests were normalized (with mean zero and standard deviation equal to one) [62]. In addition, enumerators collected year-end Chinese language and math grades given by teachers. We normalized these grades by school.

To measure non-cognitive outcomes, we used a psychological test of well-being, the Mental Health Test (MHT), to measure the mental health of children. The test is a variation of the Children’s Manifest Anxiety Scale (CMAS). The CMAS is a scale that has been widely used in the United States and other developed countries for more than a decade as a screening and clinical tool. Professor Zhou Bucheng of East China Normal University developed the MHT scale used in this study. Researchers have used this test extensively across China to measure the mental health of grade school students in urban contexts. The purpose of the test is to measure the level of each student’s anxiety. The test is scored out of 90 points, where a lower (higher) score corresponds to lower (higher) anxiety. The test results can be broken down into eight subcategories, each of which represents a specific aspect of anxiety: school performance, social relationships, loneliness, self-punishment, sensitivity, physical symptoms, fear, and impulsiveness. A score of greater than 8 on any subpart is considered clinically high and indicates a need for treatment. A total score of 65 or higher indicates high risk for mental health problems and an urgent need for professional intervention [44, 63].

Self-esteem was assessed using the Rosenberg Self-esteem Scales I & II (SES) [64]. The SES consists of 10 statements, each of which is scored as a Likert scale.

Self-efficacy was assessed using the General Self-efficacy Scale (GSE) originally created by Schwartzer and Jerusalem (1995) in the 1970s [65]. We use the Chinese language version of the GSE, developed in 1995 [66]. The scale was created to predict how individuals cope with and/or adapt after experiencing stressful events.

Table 3 shows individual and family characteristics by gender. The average age of girls and boys in our sample is 11 years old. On average, girls in our sample are slightly younger than boys (the difference is 0.07 years or 25 days; *P*-value < 0.01). About 30 % of children in our sample are from ethnic minority groups and boys in our sample are 1 % more likely to be an ethnic minority than girls (*P*-value =0.01). The average household size of girls (5.42) and boys (5.05) in our sample are significantly different (*P*-value < 0.01). More than half of the sample students have at least one parent that has migrated for work, though no significant difference is found between girls and boys (*P*-value = 0.34). Also, about half of the students’ mothers have at least a primary school education, though no significant difference was found between the genders (*P*-value = 0.72). The education level of fathers in our sample is only slightly higher than that of mothers (by 1 percentage point). In the case of the sample fathers, we do find a significant difference between boys and girls in our sample (*P*-value < 0.01), as 70 % of the fathers of girls have at least a primary school education, while 69 % of the fathers of boys do. In summary, then, although there are differences in the individual and family characteristics of boys and girls, the magnitude of these differences is rather small. In the following section, we consider whether these differences impact our estimates.

**Table 3:** Comparisons of the individual and family characteristics of girls and boys in rural areas of Western China

	Girl	Boy	*P*-value for the difference between girls and boys
Age (years)	10.95	11.02	<0.01
Ethnicity (1 = ethnic minority; 0 = otherwise)	0.31	0.32	0.01
Household size	5.42	5.05	<0.01
At least one parent takes migrant job (1 = yes; 0 = otherwise)	0.54	0.55	0.34
Education of mom (1 = at least primary education; 0 = otherwise)	0.52	0.52	0.72
Education of dad (1 = at least primary education; 0 = otherwise)	0.70	0.69	<0.01

Data source: Authors’ data

## Results

Overall, health and nutrition indicators for the full sample are poor (Table 4). The WAZ and HAZ of the sample children are -0.67 and -0.97, respectively. The STH infection rate is 41.9 %, and the anemia prevalence is 15.9 %.

**Table 4:** Outcomes for the average children in rural areas of western China

(1)	(2)	(3)	(4)	(5)
Outcome number	Outcomes	Sample size	Unit	Value
1	WAZ	1,450	Z-score	-0.67
2	HAZ	4,223	Z-score	-0.97
3	Anemia rate	50,598	%	15.87
4	STH infection rate	2,179	%	41.85
5	MHT	30,529	0–90 points	36.35
	Learning anxiety	30,529	0–15 points	7.94
6	Self-esteem	13,119	10–40 points	25.25
7	Self-efficacy	11,319	10–40 points	25.69
8	Working memory	4,305	45–150 points	78.49
9	Processing speed	4,305	45–160 points	87.33

See Table 2 for complete definitions of all variables. We have sufficient power for our tests due to the large sample size of the dataset we pooled together from seven surveys. For a two-sided test with a significance level of 0.05 and when the effect estimator has a limiting normal distribution, we have 80 % power to detect a MDE (minimum detectable effect) of 0.16 of WAZ, 0.10 of HAZ, 1 percentage points of anemia rate, 6 percentage points of STH infection rate, 0.48 points of MHT, 0.16 points of self-esteem score, 0.31 points of self-efficacy score, 0.03 SD of standardized math test score, 0.06 SD of math course grades given by teachers, 0.05 SD of standardized Chinese test score, 0.04 SD of Chinese course grades given by teachers, 0.86 points of WMI and 1.14 points of PSI [78, 79]

In this table, we only include outcome measure of variables that have standalone significance. We do not include (for example) standardized test scores of math and Chinese language scales since these scores are only used to compare the relative performance of children of different genders

Data source: Authors’ data

With the exception of anemia, there are no statistically significant differences between boys and girls in terms of health or nutrition (Table 5). Boys in our sample have slightly higher WAZ (*P*-value = 0.40) and HAZ (*P*-value = 0.27) scores, however, the differences are not statistically significant. Similarly, the STH infection rate is 3 percentage points higher among boys (43.31 %) than among girls (40.16 %), but the difference is again statistically insignificant (*P*-value = 0.14). Anemia prevalence among girls (16.3 %), however, is statistically higher than that among boys (15.5 %, *P*-value = 0.01), although the raw difference is less than one percentage point.

**Table 5:** Comparison of health, nutrition, cognition, educational status, and non-cognitive outcomes of girls and boys in rural areas of western China

(1)	(2)	(3)	(4)	(5)	(6)	(7)
Outcomes number	Outcomes	Sample Size	Unit	Boys	Girls	*P*-value
1	WAZ	1,450	Z-score	-0.64	-0.69	0.40
2	HAZ	4,223	Z-score	-0.95	-0.99	0.27
3	Anemia prevalence	50,598	%	15.46	16.32	0.01
4	STH infection rate	2,179	%	43.31	40.16	0.14
5	MHT	30529	0–90 points	35.20	37.64	<0.01
6	Self-esteem	13,119	10–40 points	25.35	25.13	<0.01
7	Self-efficacy	11,319	10–40 points	25.87	25.49	<0.01
8	Standardized math test	49168	SD	0.07 ± 0.01	-0.08 ± 0.01	<0.01
9	Math course grades given by teachers	9532	SD	-0.02 ± 0.01	0.02 ± 0.01	0.05
10	Standardized Chinese test	13,707	SD	-0.04 ± 0.01	0.04 ± 0.01	<0.01
11	Chinese course grades given by teachers	15,753	SD	-0.14 ± 0.01	0.15 ± 0.01	<0.01
12	Working memory	4,305	45–150 points	78.16	78.88	0.02
13	Processing speed	4,305	45–160 points	86.25	88.6	<0.01

See Table 2 for complete definitions of all variables. SD = standard deviation

Data source: Authors’ data

In terms of cognition and educational performance, girls outperform boys on five of the six outcome measures. Girls score higher than boys on both the working memory (*P*-value = 0.02) and processing speed scales (*P*-value < 0.01, Table 5). Girls also score higher than boys on the standardized Chinese language test (*P*-value < 0.01), and are given higher grades by teachers in both math (*P*-value = 0.05) and Chinese language (*P*-value < 0.01) classes. For the latter class, in particular, girls outperform boys by about one third of a standard deviation. On the other hand, boys (0.07 SD) outperform girls (-0.08 SD) on the standardized math test (*P*-value < 0.01).

Average sample scores from the three non-cognitive assessments; the MHT (36.35 out of 90), the self-esteem test (25.25 on a scale from 10 to 40), and the self-efficacy test (25.69 on a scale from 10 to 40) show that poor performance is common among both genders in the sample (Table 4). Girls, however, perform consistently worse than boys on all three assessments (Tables 5 and 6). Out of the three tests, the overall MHT score (Table 5) shows the largest difference between the two groups, amounting to 2.44 points (*P*-value < 0.01) between girls (37.64) and boys (35.20). Put differently, girls score 6.93 % higher than boys, indicating that they have *more* anxiety than their male counterparts. Table 6 shows the subcategories of the MHT, where the scores differences between the two genders are small, but precise.^1^

**Table 6:** Comparison of categorical breakdown of the MHT score between girls and boys in rural areas of western China

(1)	(2)	(3)	(4)	(5)	(6)
Outcome number	Outcomes	Unit	Boys	Girls	*P*-value
1	Learning anxiety	0–15 points	7.71	8.19	<0.01
2	Personal anxiety	0–10 points	3.87	4.09	<0.01
3	Loneliness anxiety	0–10 points	2.82	2.76	0.01
4	Self-Blaming tendency	0–10 points	5.03	5.42	<0.01
5	Sensitivity tendency	0–10 points	4.58	4.72	<0.01
6	Body anxiety	0–15 points	4.92	5.12	<0.01
7	Phobia anxiety	0–10 points	4.95	3.70	<0.01
8	Impulsive tendency	0–10 points	2.57	2.39	<0.01
9	Total	0–90 points	35.20	37.64	<0.01

Data source: Authors’ data

In order to check if the individual, family and geographical differences between girls and boys confound our findings regarding the gender gap, we regressed three of our outcome variables (anemia, standardized math and Chinese test scores) on the gender variable while controlling for individual and family characteristics and provincial dummies (Table 7). We find that the regression results are largely in line with the descriptive results in our paper, indicating that the individual, family and geographical differences between girls and boys do not affect the descriptive findings on the gender gap. For example, the regression results show that girls are 2 percentage points more likely to be anemic than boys (*P*-value < 0.01). This finding that there is a slightly higher anemia prevalence among girls in our sample than among boys is consistent with the descriptive analysis. The regression results also show that girls perform better than boys in the standardized Chinese test by 0.09 SD (*P*-value < 0.01), which is similar to the 0.08 SD (*P*-value <0.01) margin found from the descriptive analysis. The regression results are also consistent with the descriptive analysis in showing that boys on average score higher on the standardized math test than girls.

**Table 7:** OLS regression results of the gender gap in the health and educational outcomes

	Anemia	Standardized math test	Standardized Chinese test
	(1)	(2)	(3)
Gender (1 = male;0 = female)	-0.02*** (0.00)	0.21*** (0.01)	-0.09*** (0.02)
Age (years)	0.01*** (0.00)	-0.06*** (0.01)	-0.07*** (0.01)
Ethnicity (1 = ethnic minority; 0 = otherwise)	-0.00 (0.01)	-0.19*** (0.03)	-0.31*** (0.04)
Household size	0.00** (0.00)	-0.02*** (0.00)	-0.03*** (0.01)
Mother’s education (1 = at least primary education; 0 = otherwise)	-0.01** (0.00)	0.03*** (0.01)	0.02* (0.01)
Father’s education (1 = at least primary education; 0 = otherwise)	-0.00 (0.00)	0.16*** (0.01)	0.14*** (0.01)
At least one parent takes migrant job (1 = yes; 0 = otherwise)	0.01** (0.00)	-0.05*** (0.01)	0.00 (0.02)
Household asset (1 = higher than median; 0 = otherwise)	-0.02*** (0.00)	0.05*** (0.02)	0.01 (0.02)
Province dummies	Yes	Yes	Yes
Constant	-0.01 (0.02)	0.40*** (0.08)	0.79*** (0.10)
Observations	26,919	19,101	13,645
R-squared	0.007	0.061	0.060

Each column presents the OLS regression results from regressing the outcome variables (i.e. anemia, standardized math test scores and standardized Chinese test scores) on the gender variable while controlling for the individual variables and family variables. Provincial dummies are included in all regressions. Robust standard errors in parentheses; *** *p* < 0.01, ** *p* < 0.05

We conduct a few tests to examine the robustness of the above results. First, in order to check whether the gender difference is sensitive to student age, we compare the regression of gender gap for the whole sample students (Table 7) with the regression including only the students of age 10 to 12, who constitute the majority of the sample (Table B1 in Additional file 1). We find that excluding the students aged under 10 and over 12 almost does not change the estimated gender gap.

Second, to assess whether the estimated gender gap is sensitive to the wealth levels of families, we run two types of regressions to estimate the gender gap: one with and one without controlling for the family’s level of household assets (Table 7 and Table B2 in Additional file 1). We find that controlling for household assets (versus not controlling for household assets) does not change the estimated gender gap.

Finally, we examine whether the gender difference comes from across or within provinces. We compare the regression of gender gap including the provincial dummies (Table 7) with the regression without the provincial dummies (Table B3 in Additional file 1) and find that the gender gap we observe comes from within provinces instead of across provinces. For more details on any of these tests, please refer to Additional file 1.

In summary, girls have the same nutritional and health outcomes as boys, except for anemia. In academics, except for standardized math score, girls do better than boys. The gender gap, however, is still very much evident when it comes to non-cognitive outcomes. Girls in our sample have lower self-esteem, lower self-efficacy and more overall anxiety than their male counterparts. Further analysis shows that, overall, our estimates are robust; the evidence for the narrowing gender gap is not sensitive to the removal of the relatively younger and older students from our sample. Neither is the evidence sensitive to the level of household assets or provincial heterogeneity. These robustness checks further suggest that the gender gap in China has indeed become modest.^2^

## Discussion

The primary aim of this study has been to document whether rural Chinese girls are worse off than their male counterparts in terms of health, nutrition, cognition, educational performance, and non-cognitive outcomes. Overall, our findings suggest that with a few exceptions, the gender gap in our study areas in China appears to be modest.

We appear to be documenting a shifting pattern in China. In studies in the 1990s and early 2000s, many research teams documented gender gaps in the terms of many health, nutrition and cognitive variables. However, in our study (using data from 2008 to 2013), the gender gap among school-aged children, as it pertains to physical health and academic performance, appears to be taking a similar trajectory to that in developed countries. With the exception of non-cognitive outcomes, anemia and standardized math test scores, the gender gap in our study areas in China appears to be diminishing. While it is beyond the scope of our paper to identify the exact mechanism behind this shift, it may be due to some combination of rising incomes [67–69], falling levels of fertility, and better employment opportunities off the farm [70]. The evidence suggests that Chinese families, even those in relatively poor rural areas, are treating girls and boys on a more equal basis.

Even more interestingly, the gender gap for cognition and educational performance has not only disappeared, but has also reversed. Our data show that girls perform better on standardized tests of cognition and Chinese language than do boys, and also receive higher grades in math and Chinese language classes. The only academic area in which boys still outperform girls is on standardized math tests. On the one hand, our standardized math test results are consistent with the literature base that shows that boys outperform girls in math, even in the case of developed countries [71, 72]. Explanations for why boys tend to perform better in math are complicated, but it is commonly thought that societal expectations may exert an influence on the relative academic performance of male and female students. On the other hand, a possible reason for the differences between the standardized math test gender gaps and the teacher-graded math test gaps may be that the results reflect the teachers’ own biases and their expectations of students. Studies have shown that when teachers grade student tests, scoring is likely to be affected by their perceptions of student performance or their preferences toward male or female students [73]. For example, if teachers perceive that girls are higher-performing students or if they favor female students, girls may receive higher marks than their actual performance on a test deserves.

These gains, however, may have come at a cost. Girls are lagging behind boys in terms of their non-cognitive outcomes. Girls have higher levels of learning anxiety and lower levels of both self-esteem and self-efficacy. Our results are consistent with the international literature which finds that girls are more likely to suffer from mental health problems, to exhibit higher levels of depression or anxiety and to have lower levels of self-esteem during adolescence than boys. In summary, our results show that girls outperform (or exhibit no significant differences from) boys in most health and educational outcomes with the exception of a number of non-cognitive outcomes.

This study has a number of strengths. First, the large size of the aggregated sample, comprising seven different datasets, is much larger (n > 69,000) than that of similar studies. This gives the study a high degree of statistical power and considerable external validity—at least for relatively poor and rural regions of western China. Second, all of the observations were collected by a single research team that used a common sampling strategy. The data collection instruments and the enumeration protocols were both standardized, allowing us to take advantage of the full aggregated sample in our analyses.

The study has several limitations. First, given the nature of the sample, it is not possible to extrapolate our findings to China’s non-poor areas. However, given the fact that our study areas are among the poorest in China, and the fact that the international literature suggests that the gender gap is narrower in richer areas, we can be fairly assured that the overall status of girls in China has improved relative to that of boys over the past decade. Second, although the paper compares girls and boys on a number of different outcomes, we are unable to identify the exact cause of any observed differences. Third, although most outcomes measures are derived from datasets collected in multiple provinces, the data for some outcome measures (such as WAZ, HAZ, Working memory index and Processing speed index) are only available from one province (Guizhou Province). As a result, the findings for these outcomes measures may have limited generalizability to other areas of China.

The results in this paper should not be construed to mean that children in China’s poor areas are not vulnerable. Indeed, the absolute levels of health, nutrition, cognition, educational performance, and non-cognitive outcomes among both girls and boys are still low. In comparison with international standards, children in rural China—both boys and girls—are shorter and lighter. Over 40 % are infected with intestinal worms. Other work has documented the poor levels of educational performance among rural children relative to urban children [74]. Perhaps a more accurate interpretation of the results of this paper is that *all* children in the rural areas of western China are vulnerable. They all require extra care, attention and resources.

From a policy perspective, our results may indicate the success of existing programs that aim to improve outcomes for rural Chinese girls. Additional research should focus on measuring the impact of such programs. If positive impacts from such programs can be identified, perhaps such programs should be expanded to cover boys as well as girls.

Increasing the number of female students who complete higher levels of education and achieving greater gender equality in education is important for boosting female labor force participation [75]. Improving female labor market outcomes is part of a process that can ensure strong, sustainable, balanced economic growth for China in the future. Beyond economic efficiency, higher levels of gender equality in education and in the workforce can contribute to eliminating discriminatory social institutions and cultural norms, and in turn increase the happiness and well-being of women and society as a whole [76]. Although our results show that rural areas in western China has made huge progress toward narrowing the gender gap in childhood and adolescence, gender inequalities still persist. For example, the fields of study typically chosen by young men and women appear to be perpetuating gender labor market segregation [77]. The government has an important role to play in promoting gender equality both in schools and in the workforce.

## Conclusion

Our findings suggest that with the exception of non-cognitive outcomes, anemia and standardized math test scores, the gender gap in our study areas in China appears to be diminishing. With the exception of anemia rates and mental health indicators, the health outcomes of girls are equal to those of boys. Girls and boys are statistically identical in terms of weight-for-age, height-for-age, and prevalence of intestinal worm infections. Girls performed better than boys on five of six cognitive and educational performance indicators. With the exception of math standardized test score, girls score higher than boys on the working memory, the processing speed scales, the standardized Chinese language test, math and Chinese grades given by teachers.

To our knowledge, this paper is one of the first studies to document a shifting pattern of gender gap in rural China by using large-scale first-hand survey data. In studies in the 1990s and early 2000s, many research teams documented gender gaps in the terms of many health, nutrition and cognitive variables. However, in our study (using data from 2008 to 2013), the gender gap among school-aged children, as it pertains to physical health and academic performance, appears to be diminishing and taking a similar trajectory to that in developed countries.

## Abbreviations

CMAS: Children’s manifest anxiety scale
GDP: Gross domestic product
GSE: General self-efficacy scale
HAZ: Height-for-age z-scores
Hb: Hemoglobin
MHT: Mental health test
PPP: Purchase power parity
PSI: Processing speed index
SES: Rosenberg self-esteem scales I & II
STH: Soil-transmitted helminth
WAZ: Weight-for-age z-scores
WHO: World Health Organization
WISC-IV: Wechsler Intelligence Scale for Children, Fourth Edition
WMI: Working memory index

## Footnotes

1 In order to check whether the gender difference is sensitive to the ethnic minority status, we compare the outcomes between the two gender groups excluding ethnic minorities from our sample (Table B4 in Additional file 1). We find that girls are still not statistically significantly different from boys in terms of WAZ, HAZ and STH infection rates. Also, girls are slightly more likely to be anemic than boys (*P*-value < 0.01), outperform boys on five out of the six education and cognition outcome measures, and perform worse than boys on all three non-cognitive outcomes, outcomes that are all consistent with results for the whole sample in Table 5. Because of this, we do not believe that ethnicity is a factor affecting the results on gender gaps among our sample.

2 By looking at gender differences in our outcomes across years, we show that the patterns in the gender gap over time are largely consistent with the analytical results of our paper. For example, girls score higher than boys on the standardized Chinese language test (*P*-values < or =0.01) in 2011 and 2012. Boys outperform girls on the standardized math test in all years from 2009 to 2012 (*P*-values < 0.01). Additionally, girls tend to perform worse than boys in non-cognitive outcomes. For example, girls score higher than boys on the Mental Health Test from 2009 to 2011, indicating girls are more anxious than boys (*P*-values <0.01). Also, girls, on average, had lower measures of self-esteem than boys from 2010 to 2012 (*P*-values < or =0.01). Consistent with the results in the paper, anemia levels are higher for girls than for boys in 2011 and 2012 (*P*-values <0.05), although the difference is not as significant as in the years from 2009 and 2010.
